# The complete chloroplast genome sequence of *Gomesa flexuosa*

**DOI:** 10.1080/23802359.2022.2093670

**Published:** 2022-07-07

**Authors:** Ping Mo, Jinhua Zhou, Fumin Zhou, Yazhi Chen, Kerui Huang

**Affiliations:** College of Life and Environmental Sciences, Zoology Key Laboratory of Hunan Higher Education, Hunan Provincial Key Laboratory for Health Aquaculture and Product Processing in Dongting Lake Area, Hunan Provincial Key Laboratory for Molecular Immunity Technology of Aquatic Animal Diseases, Changde innovation team of Wetland Biology and Environmental Ecology, Hunan University of Arts and Science, Changde, China

**Keywords:** *Gomesa flexuosa*, chloroplast genome, phylogenetic analysis

## Abstract

*Gomesa flexuosa* (Lodd) M.W.Chase & N.H.Williams is a new species of orchid revised in 2009. In this study, the chloroplast genome of *G. flexuosa* was sequenced to determine its genomic characteristics and phylogenetic relationship with other related species. *G. flexuosa* has a chloroplast genome size of 147,764 bp, comprising 25,757 bp of two inverted repeat (IR) regions, 83,579 bp of large single-copy (LSC) region, and 12,671 bp of small single-copy (SSC) region. Moreover, the whole genome contains 73 protein-coding genes, 38 tRNA genes, and eight rRNA genes. Phylogenetic analysis showed that *G. flexuosa* is closely related to *Oncidium sphacelatum*.

*Gomesa flexuosa* (Lodd.) M.W.Chase & N.H.Williams was first published as *Oncidium flexuosum* in 1820 (Loddiges 1820) and revised as *G. flexuosa* in 2009 (Chase 2009). The species belonging to the orchid family is native to Argentina, Brazil, and Paraguay (Chase 2009). However, it is currently cultivated worldwide because of its economic importance as a cut flower species. *G. flexuosa* has also been crossed with closely related species to breed new varieties (Li et al. 2021). The development of new breeding strategies relies on understanding the phylogenetic relationships, genetic diversity, and genetic structure of species. Phylogenetic tree reconstruction by several cpDNA and nrITS fragments indicated that *Gomesa radicans* is closely related to *G. flexuosa* (Neubig et al. 2012). However, the chloroplast genome of *G. flexuosa* has not been sequenced, despite its potential role in providing more genetic variations information to infer species relationships. In this study, the complete chloroplast genome of *G. flexuosa* was sequenced, assembled, and annotated to provide useful genomic resources for future studies on the phylogenetic relationship of Orchidaceae and the breeding of new varieties.

Fresh leaves were obtained from *G. flexuosa* cultivated in Baima Lake of Changde, Changde, Hunan province, China (N29°03′6.48″, E111°40′00.10″, 33 m). The voucher specimens were preserved at the College of Life and Environmental Sciences, Hunan University of Arts and Sciences (contact person: Ping Mo, moping2015@126.com, voucher number MP0186). Total genomic DNA was extracted from the leaves using a DNeasy plant tissue kit (TIANGEN Biotech Co., Ltd., Beijing, China). Genome skimming sequencing was performed on Illumina Hiseq 2500 platform. A total of 67,962,540 reads were retained after filtering out the low-quality reads using fastp (Chen et al. 2018). Subsequently, *de novo* assembly of the *G. flexuosa* chloroplast genome was performed using GetOrganelle v1.7.5 (Jin et al. 2020), and 5,000,000 reads were randomly selected to reduce the computational time and resources. Plastid Genome Annotator (PGA; Qu et al. 2019) was used to annotate the chloroplast genome.

The chloroplast genome of *G. flexuosa* is a circular molecule with a length of 147,764 bp. It has a typical quadripartite structure containing a large single-copy (LSC, 83,579 bp) region, a small single-copy (SSC, 12,671 bp) region, and two inverted repeat regions (IRs, 25,757 bp). The overall GC content of the chloroplast genome is 37.13%, while the GC contents of LSC, SSC, and IR regions are 34.55%, 23.74%, and 43.43%, respectively, of the entire genome. A total of 119 genes were annotated in the chloroplast genome, including 73 protein-coding genes, 38 tRNA genes, and eight rRNA genes.

Maximum-likelihood (ML) tree was constructed using IQ-Tree v1.6.12 (Chernomor et al. 2016) based on the chloroplast genome of *G. flexuosa* and its related species to determine the phylogenetic position of *G. flexuosa*. A total of 40 chloroplast genomes were downloaded from GenBank and aligned using MAFFT v7.4.75 (Rozewicki et al. 2019). The node supports were evaluated through 1000 fast bootstrap replicates. The results showed that *G. flexuosa* is closely related to *Oncidium sphacelatum* Lindl. with 100% of the support (Figure 1). The phylogenetic tree revealed that the genus *Oncidium* is a paraphyletic group, inconsistent with the phylogenetic relationship previously inferred from multiple cpDNA and nrITS sequence fragments (Neubig et al. 2012). Therefore, the phylogenetic relationship between genera *Oncidium* and *Gomesa* should be studied further based on genetic variation at the genome level.

**Figure 1.**
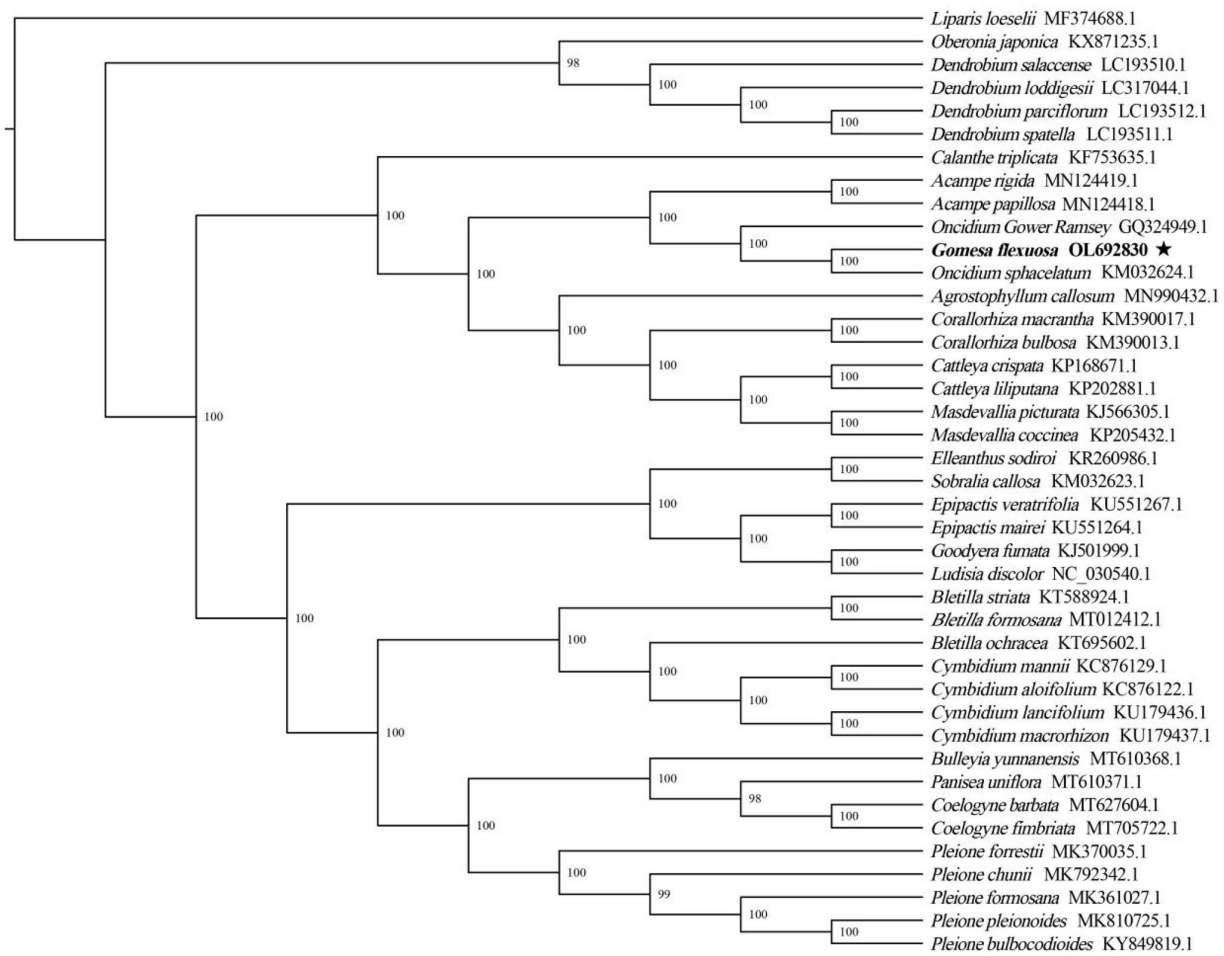
Maximum-likelihood (ML) tree of *Gomesa flexuosa* and 40 relative species was reconstructed using the IQ-Tree based on complete chloroplast genome sequences. Bootstrap values are shown next to the nodes.

## Data Availability

The complete chloroplast genome sequence of *Gomesa flexuosa* has been deposited in the GenBank database under the accession number OL692830 (https://www.ncbi.nlm.nih.gov/nuccore/OL692830). The associated BioProject, SRA, and Bio-Sample numbers are PRJNA807811, SRR18192490, and SAMN26001590, respectively.
